# Molecular Alterations in Metastatic Ovarian Cancer From Gastrointestinal Cancer

**DOI:** 10.3389/fonc.2020.605349

**Published:** 2020-12-10

**Authors:** Chao Chen, Xiaoxu Ge, Yamei Zhao, Da Wang, Limian Ling, Shu Zheng, Kefeng Ding, Jian Wang, Lifeng Sun

**Affiliations:** ^1^ Department of Colorectal Surgery and Oncology, Key Laboratory of Cancer Prevention and Intervention, Ministry of Education, The Second Affiliated Hospital, Zhejiang University School of Medicine, Hangzhou, China; ^2^ Department of Cancer Institute, The Second Affiliated Hospital, Zhejiang University School of Medicine, Hangzhou, China

**Keywords:** metastatic ovarian cancer, gastrointestinal cancer, molecular alteration, metastatic pathway, metastatic mechanism

## Abstract

Reports indicate that most metastatic ovarian cancer (MOC) originates from gastrointestinal cancer (GIC). Notably, GICs metastasize to the ovary frequently *via* 3 main routes including hematogenous spread, lymphogenous spread, and transcoelomic spread. Nonetheless, the mechanism of the progression remains unknown, and only a handful of literature exists on the molecular alteration implicated in MOC from GIC. This work collected existing evidence and literature on the vital molecules of the metastatic pathway and systematically analyzed them geared toward exploring the mechanism of the metastatic pathway of MOC. Further, this review described dominating molecular alteration in the metastatic process from cancer cells detaching away from lesions to arrive at the ovary, including factors for regulating signaling pathways in epithelial-interstitial transformation, invading, and surviving in the circulatory system or abdominal cavity. We interrogated the basis of the ovary as a distant metastatic site. This article provides new insights into the metastatic pathway and generates novel therapeutic targets for effective treatment and satisfactory outcomes in GIC patients.

## Introduction

Ovarian carcinoma is one of the most prevalent cancers among the female population, and the second leading lethal cause of gynecologic malignancies (1). Metastatic ovarian cancer (MOC) accounts for up to 2.3% to 23.7% of all malignant ovarian cancers that are usually transferred from different organs (2, 3). A recent statistic study in Japan discovered that MOC frequently originates from the gastrointestinal (GI) tract (71%), followed by the appendix (8%), breast (6%), and pancreas (4%) (4). MOC is unique compared to other gynecologic malignancies with non-obvious symptoms when it is in the early stages (an abdominal mass and/or fullness is the common symptom) and no special findings detected *via* imaging analysis (5). MOC from GIC develops rapidly and often affects young women with obvious symptoms developing in the later stage. Meanwhile, it is relatively chemoresistant (6). Because of better prognosis, many researchers recommend surgery and hyperthermic intraperitoneal chemotherapy (HIPEC) as therapeutic interventions; however, the median survival period ranges only from 19 to 27 months. Recurrence and distant metastasis still exist after the detection (7). As such, it is necessary to investigate the mechanism of MOC from GIC for improving the survival rate and quality of life among the patients.

Researchers have fronted various speculations on how GIC metastasizes to the ovary, notably, transcoelomic spread is the most classic because of the coincidence with peritoneal metastasis. Scholars further reveal that the cancer cells first penetrate the serosa layer, fall off into the abdominal cavity or ascites, and eventually implant into the ovarian envelope by peristalsis of intestine and gravity. Nevertheless, this hypothesis remains unconvincing to the clinicians since the infiltration depth of the GIC did not reach the serous layer. Besides, MOC were located in the ovarian stroma rather than on the surface of the ovary, and the capsule was intact in part of MOC patients from GIC. Notably, accumulating evidence recommend hematogenous spread and lymphogenous spread. For instance, Yamanishi Y et al. revealed that hematogenous spread is the prominent pathway in MOC from colorectal cancer (CRC) due to 67% of MOC patients from CRC with vascular invasion (8). However, other scholars insisted that it frequently disseminates *via* lymphogenous pathway with the existence of mucosal and submucosal lymphatic plexus. Additionally, GIC infiltrates retroperitoneal lymph nodes, which trigger lymphatic obstruction, then lymphatic reflux in the ovaries (9). The combination of these metastatic routes appears to occur in various situations, particularly in advanced GIC (8).

While acknowledging the progress made in the mechanism of metastasis, information on the MOC remains scant. The mechanism of tumor cells detaching from the primary site into the circulation, surviving in the circulatory system or abdominal cavity, and invading and growing in the ovary have always been elusive to the clinicians and the research of the tumor. Therefore, a better understanding of the metastatic process vitally contributes to effectively treating MOC from GIC. Here, we review existing studies to explore the potential metastatic pathway and molecular alterations of MOC from GIC.

## Cell Detachment

Different metastatic pathways have the same initiate—cell detachment. First, GIC cells detach from primary cancer and then invade into the surrounding tumor-associated stroma. While the mechanisms of detachment remain unclear, this process is widely thought to occur due to altered cell adhesion. In recent years, integrins, as transmembrane glycoproteins, have attracted increasing attention due to their roles in mediating adhesion between the actin cytoskeleton and specific extracellular matrix ligands (10). Integrin α2β1 has been reported to be implicated in the GIC metastasis process (11). Wu et al. reported higher expression of integrin α2β1 in patients with metastasis after detecting the expression of integrin α2β1 both in the GIC patients with or without metastasis. Further, they knocked down the integrin β1 in HCT 116 cells and found that the ability of migration and invasion of these cells was lower compared to the wild type HCT 116 cells both *in vitro* and *in vivo*. This confirms that integrin α2β1 is vital in CIG metastasis and designed that integrin α2β1 might be an innovative strategy for GIC therapy (11). Additionally, matrix metalloproteinases (MMPs) including meprin α and meprin β abundantly located in intestinal enterocytes are key in cell detachment. Meprin α was earliest discovered in the CRC cell line Caco-2 (12). It promotes the epidermal growth factor receptor/mitogen-activated protein kinase (EGFR/MAPK) signaling pathway by dividing into epidermal growth factor (EGF) and transforming growth factor α (TGFα). As a result, it induces the proliferation and migration of Caco-2 cells (13). Notably, the epithelial adhesion protein E-cadherin is a substrate of meprin β. Downregulated meprin β induces separating E-cadherin and cancer cells detaching from cancer (14). Recent studies have demonstrated that upregulated expression of MMP-2, MMP-7, MMP-9, and MT1-MMP trigger GIC metastasizing to distant organs (15–17). As such, regardless of the route the tumor cells enter the stroma, there is a significant opportunity for subsequent metastasis.

## Metastatic Route

### Hematogenous Spread

Other researchers agree that the hematogenous spread is the primary mechanism of GIC metastasis to the ovary (18–20). Further evidence reveals that (a) the ovary is rich in blood vessels with frequent cancer embolus and (b) MOC from GI are usually detected in young women. Yibin Cai et al. analyzed 154 patients with MOC from GIC and concluded 43.5 years as the median age of onset, and the patients were premenopausal. Patients younger than 50 years old had independent risk factors (21); (c) 87.4% MOC with a primary tumor of the GI tract had bilateral involvement of the ovaries (22).

In hematogenous metastases, GIC cells invade into the blood vessel. On one hand, a few molecules promote GIC cells to cross the microvessels. A recent study showed that E-cadherin effects via Wnt signaling, Rho GTPases, and epidermal growth factor receptor (EGFR) in the process (23). On the other hand, MOC was promoted by growing new cancer blood vessels from the existing vascular network, thereby expanding the contact area of the cancer blood vessel system, and forming vessels that are inadequate in both morphology and function. Recent studies indicated that the lumen of tumor blood vessels is partly composed of cancer cells (24). However, the new cancer blood vessel is tortuous and easy to leak, and it can continuously restructure their morphology (25). In hypoxia, c-Myc represses miR-15-16, which induces angiogenesis and distant metastasis of solid tumors by downregulating fibroblast growth factor 2 (FGF2) in the Wnt pathway (26). Notably, MALAT1 belongs to long non-coding RNAs (lncRNAs), which play critical roles in the hematogenous spread of GIC (27). Based on Li et al., MALAT1 induces angiogenesis by increasing the expression of β-catenin and E-cadherin as well as through the ERK/MMP and FAK/paxillin signaling pathways (28). Also, MALAT1 altering angiogenesis was associated with miR-126, which is an intron of the EGFL7 gene. MALAT1 cooperates with miR-126, altering the acetylation level of H3 histone in the EGFL7 promoter region to boost the EGFL7 expression level and hence promote the hematogenous spread of GIC (29, 30). Wang et al. established that the lncRNA UCA1 increases the degradation of GRK2 *via* Cbl-c-mediated ubiquitination following the activation of the ERK-MMP9 pathway, which might be involved in vascularization (31). YUUKI IIDA et al. revealed the expression of PS-PLA1 is associated with GIC hematogenous spread. The potential way might be that PS-PLA1 stimulates the PTX-sensitive and Ki16425-sensitive cell surface receptors (32). Importantly, vascular endothelial growth factor (VEGF) enhances angiogenesis. VEGF-A, VEGF-B, and placental growth factor (PIGF) stimulate blood vessel growth by binding to VEGFR-1. A research reviewed 91 GIC tissue including non-metastasis, lymphogenous spread, and hematogenous spread to explore the role of VEGF-B in metastasis. The results showed that VEGF-B exist both in endothelial and tumor cells regardless of the metastatic status. Over-expression of VEGF-B was implicated in hematogenous spread (p=0.006) (33). Morever, VEGF was dramatically associated with the progression and hematogenous metastasis of GIC (34).

### Lymphogenous Spread

Notably, lymphogenous spread is another prominent pathway for GIC (35, 36). One study showed that MOC is related to retroperitoneal lymph node relapses. They reviewed 105 CRC patients with PM who underwent CRS plus intraperitoneal chemotherapy and found retroperitoneal lymph node relapses in 19 patients including 18 with OM and 1 without OM (p=0.001). Only 1% of CRC patients after surgery had retroperitoneal lymph node recurrence, but it accounts for 29% of CRC patients with OM in this study. OM is the only predictive factor for retroperitoneal relapse (p=0.0012) (37).

Of note, the nature of lymphatic vessels is easier for metastasis compared to blood vessels. The inter-endothelial junction is not tight with no basement membrane around the lymphatic vessels (38). Furthermore, lymphatic vessels provide more appropriate flow velocity and shear stress for survival of GIC than the bloodstream. Another explanation is the anatomy. Lymphogenous spread exists at the early stages of GIC due to the rich lymphatic plexus (38). The urogenital lymph vessel tract generates the receptaculum chyli *via* the lumbar trunks. The receptaculum chyli joins intestinal trunks, to which the gastric, hepatic, pancreaticolienal, and mesenteric (superior mesenteric and mesocolic) nodes are connected *via* celiac lymph nodes. With the short distance between the receptaculum chyli and the gastric nodes, it is effortless for the metastasis of GC cells to the urogenital lymph vessel trunks, which provide the ovaries (8). Moreover, GIC cells probably obstruct lymphatic vessels then the countercurrent of the cancer cell flow into the ovaries occurs.

Due to the absence of appropriate molecular markers, the exact mechanism of lymphogenous spread remains unknown (39). In recent studies on lymphogenous spread, VEGF-C and VEGF-D are widely used because of their abundance in intratumoral lymphatic vessels. Additionally, VEGF-C and VEGF-D belong to the VEGF family and stimulate the growth and migration of lymphatic vessels *via* VEGFR-3, which expresses in lymphatic endothelial cells (40). Theoretically, VEGF-C and VEGF-D are likely to promote metastasis in 3 mechanisms, i.e. (1), enlarging the surface area where cancer cells contact the lymphatic endothelium (2); facilitating vascular permeability; and/or (3) changing the adhesive properties or cytokine/chemokine expression of the lymphatic endothelium. Moreover, the factors mentioned above change the cancer interstitial fluid pressure, which determines cancer cell seeding. Noteworthy, lymphogenous spread is enhanced by attracting CCR7-positive tumor cells to secondary lymphoid tissue chemokine (SLC)-expressing lymphatic endothelium (41). One hypothesis suggested that cancer cells limited to grow in certain space cause mechanical stress to compress the newly formed lymphatic channels inside the cancer, whereas, at the periphery, lymphatics are wider due to excess VEGF-C. Enlarged lymphatics probably gather interstitial fluid and metastatic cancer cells, which “ooze” from the cancer surface to promote lymphatic metastasis. Functional lymphatics within cancers is less or absent, and thus it does not merely result in interstitial hypertension, but hinders the delivery of therapeutic agents (42). It is also likely that lncRNA, C21orF96 lead to lymphogenous spread of GIC. Yang et al. found that C21orF96 was upregulated in positive lymph node tissues and GIC compared to normal tissues. C21orF96 elevated lymphangiogenesis of GIC by increasing the formation of tubulars, intersecting nodes, and the length of the tubes in human umbilical vein endothelial cells (HUVECs) (43).

Of note, podoplanin is a novel molecule in cancer research. It is a small mucin-type transmembrane glycoprotein highly expressed in mouse colon adenocarcinoma. Raica M et al. revealed that overexpression of podoplanin in cancer cells was prone to invade and metastasize because podoplanin is implicated in poor clinical outcome of patients (44). According to Leah N. Cueni et al., podoplanin does not promote the growth of cancer but readily facilitates the movement of cancer cells to other sites. They claimed that the expression of podoplanin is mediated by the molecules that existed in the tumor stroma, including endothelin-1, villin-1, and tenascin-C. Interestingly, podoplanin-positive cancer cell also overexpresses endothelin-1 (ET-1), a promoter of lymphangiogenesis, which supposedly functions by combining ENDRB that expresses on endothelial cells. The serum levels of ET-1 significantly rose in patients with lymphogenous metastasis compared to patients without lymphogenous involvement (45).

### Transcoelomic Spread

Transcoelomic spread is considered as a passive process. There is a natural flow in peritoneal fluid to lubricate the abdominal cavity, and as a result, cancer cells can arrive at other organs (46). Transcoelomic spread is closely correlated with the formation of malignant ascites and can be confirmed by detecting cancer cells in ascites (47). When invading the serosal layer or tissue, the GIC cells can be easily scattered into the peritoneal cavity and transported *via* ascites or peritoneal fluid before seeding intraperitoneally. As such, the occurrence of transcoelomic spread is dependent on the gravity and the location of organs. Besides, GIC cells in ascites or peritoneal fluids flow to the ovary readily due to gravity (48). A famous example is the Krukenberg tumor. German pathologist Friedrich Ernst Krukenberg first discovered the Krukenberg tumor in 1896 while the true metastatic nature of the Krukenberg tumor was exposed in 1902. Krukenberg tumor exhibits various characteristics, including involving stroma, existing mucin-producing neoplastic signet ring cells, and proliferating ovarian stromal sarcomatoid. Most KT tumors metastasize from GIC (49). Because omentum and ovarian epithelium belong to the same lineage with similar biological behavior and response to treatment in CRC patients with MOC or peritoneal metastasis (PM), scholars suggest that MOC should be recognized as part of the PM spread (46). In research on the outcome of PM patients with and without ovarian metastasis, the overall survival time of GIC patients with MOC was not different from that of patients without MOC when they accepted CRS-HIPEC (50). They suggested that MOC might be the performance of peritoneal spread from GIC because of the similar biological behavior and the high coincidence of PM with MOC, which makes sense because the peritoneum and ovaries are often recognized as a continuum particularly in the context of primary cancer of these organs (51).

## Survival in the Metastasis

When GIC cells invade the circulatory system or abdominal cavity, cancer cells disseminate widely *via* the flow of fluid. However, they encounter various obstacles before arriving at the ovary, including matrix detachment, hemodynamic shear forces, and immune systems, regardless of the metastasis pathway (52). Matrix detachment means GIC cells cannot attach themselves to ECM components, which is crucial for cell survival. As a result, it will trigger GIC cells undergoing anoikis, a form of programmed cell death (53). Douma et al. found that TrkB, a neurotrophic tyrosine kinase receptor, can suppress the anoikis of rat intestinal epithelial cells (54). TrkB promotes aggregation of cells, survival, and proliferation in suspension. When these large cellular aggregates are injected into mice, they form fast-growing tumors that metastasize to other organs. They suggested TrkB suppresses anoikis by activating the phosphatidylinositol-3-OH kinase/protein kinase B pathway. Integrins and apoptosis modulators also resist anoikis (55). Based on Jie Huang et al., Claudin-1 increases the level of membrane β-catenin, which regulates cell-cell adhesion by re-activating Akt and Src signal pathways in GIC (56). Also, hypoxia-induced ANGPTL4A in GC cells resist anoikis by activating ANGPTL4A-dependent FAK/Src/PI3K-Akt/ERK pathway and subsequently increasing PM in scirrhous GC cells (57). GIC cells and platelets form large emboli to prevent anikis. Their association, regulated by tissue factor and/or L- and P-selectins in the GIC cells, induces EMT in cancer cells (58). The combination of platelets and GIC cells help GIC cells prevent the injury of hemodynamic shear forces and the detection of immune cells in the vessels (59).

Another way cancer cells fight against the immune system is recruiting regulatory T cells (Treg), which suppress autoreactive T cells. An investigation reviewed that a large number of Treg cells in the ascitic fluid and tumor-specific T-cell could not kill cancer cells due to the existence of Treg cells (60). Elsewhere, pro-tumor T-cell suppressor cytokine phenotype of monocytes and macrophages, which do not trigger anti-tumor activity, exist in ascites and peritoneal compartments (61).

## Basis of Ovarian Metastasis

As documented, younger female GIC patients at the ovulatory phase (<40) are more likely to have MOC compared to older female patients (62). Investigators suggested that the ovulatory cycle of the ovary provides a suitable microenvironment for GIC cells to survive and invade (63). With the accumulation of steroid hormones, the epithelium of the ovary is disrupted when an oocyte is released to repair the surface of the ovary after ovulation. The process is similar to wound healing, which needs to generate new blood vessels (46). Additional articles have reported that all the isoforms of VEGF–A exist in the ovary, while both VEGFR-1 and -2 are expressed abundantly in ovarian capillaries (64). Notably, angiopoietin-2 was detected to be expressed in the ovary (65). In addition, the expression of angiogenic peptides was influenced by various parameters, including oxygen saturation, aging, and endocrine. The gonadotropic hormones, including luteinizing hormone (LH) and follicle-stimulating hormone (FSH), were detected in the ovary. LH and FSH regulate the angiogenesis in the ovary by increasing the level of VEGF in a dose-dependent manner (66). Furthermore, LeCouter J et al. found the endocrine gland–derived VEGF, and the first tissue-specific angiogenic molecule in an ovarian tissue (67). In addition to angiogenesis in the ovary, other factors stimulate GIC cells to grow, seed, invade, and survive. Cyclooxygenases (COX) could transfer to eicosanoids, which have been confirmed to improve the transformation and proliferation of GIC cells. Also, COX is associated with the presence of VEGF, which has been discussed above. COX-1 expression was highly observed in both normal or cancerous ovarian tissue, and there were lots of VEGF in the same regions. These findings indicate that COX-1 might potentially promote neovascularization and cell proliferation (68). Other growth factors, including epidermal growth factor (EGF), hepatocyte growth factor (HGF), and TGF, also regulate GIC cells metastasizing to the ovary (69). In summary, the angiogenesis caused by ovulatory cycle, the existence of COX-1, and abundance of growth factors provide a suitable microenvironment for implantation of GIC cells.

## Conclusion

In recent years, molecular alteration has been investigated to explore the importance of stromal cells and the microenvironment of GIC metastasis. Nevertheless, several fundamental questions concerning the mechanisms of GC metastasis remain unanswered. In this review, we described a metastatic process from cancer cells detached away to arrive at the ovary and established how the ovary provides a suitable situation for cancer cell survival. Based on the existing studies on the mechanism of MOC from GIC, integrins, microRNAs, and MMP are dominant factors in cell detachment. Besides, 3 possible pathways of metastasis have been suggested, including hematogenous spread, lymphogenous spread, and transcoelomic spread. Scholars widely agree that GIC metastasizes to the ovary not just *via* an isolated pathway but by the combination of these metastatic pathways. Additionally, TrkB and Treg cells help the survival of cancer in the cell circulatory system or abdominal cavity. The ovulatory cycle of the ovary provides a suitable microenvironment for GIC cells to survive and invade. Therefore, the mechanism of MOC from GIC merits comprehensive investigation, and these potential targets might be a novel approach in curing MOC from GIC.

## Author Contributions

Study concepts: LS, CC; Study design: LS, JW; Data acquisition: YZ, XG; Manuscript preparation: CC, XG; Manuscript editing: LS, JW; Manuscript revise: DW, LL, SZ, KD; Manuscript review: LS, JW. All authors contributed to the article and approved the submitted version.

## Funding

The study was funded by National Natural Science Foundation of China (No. 81472819, No. 81672342), the Zhejiang Provincial Key R&D Program of China (No. 2019C03018), the Zhejiang Provincial Natural Science Foundation of China (No. LY20H160038), and the Fundamental Research Funds for the Central Universities (No. 2019QNA7028, No. 2019FZJD009).

## Conflict of Interest

The authors declare that the research was conducted in the absence of any commercial or financial relationships that could be construed as a potential conflict of interest.
